# Concurrent and longitudinal associations between the Home Literacy Environment and the language skills of children with Down syndrome

**DOI:** 10.3389/fpsyg.2026.1795715

**Published:** 2026-04-28

**Authors:** Amelia Hickman, Rebecca Baxter, Kirstie Hartwell, Emma Pagnamenta, Vesna Stojanovik, Susan J. Loveall, Kelly Burgoyne

**Affiliations:** 1 Department of Educational Psychology, Manchester Institute of Education, University of Manchester, Manchester, United Kingdom; 2 School of Psychology and Clinical Language Science, University of Reading, Reading, United Kingdom; 3 Department of Special Education and Communication Disorders, University of Nebraska-Lincoln, Lincoln, NE, United States

**Keywords:** Down syndrome (DS), Home Literacy Environment (HLE), language, nonverbal IQ (NVIQ), shared book reading (SBR)

## Abstract

**Introduction:**

Substantial research demonstrates a positive association between the Home Literacy Environment (HLE) and vocabulary growth in typically developing (TD) children (Lovčević, 2025). Though few studies have explored this in children with Down syndrome (DS), limited evidence suggests that an enriched HLE also promotes receptive vocabulary growth in this population (Dulin et al., 2023). However, the extent to which the HLE influences expressive vocabulary development within this population remains unclear (Dulin et al., 2023).

**Research questions:**

(1) How do parent(s) of children with DS describe their HLEs, in terms of richness and (2) child engagement during shared book reading (SBR) interactions? Do these measures of the HLE (richness and child engagement) correlate with child language scores (3) concurrently and (4) longitudinally?

**Method:**

Participants were parents and children with Down syndrome (DS) aged 2 years 11 months – 6 years 10 months at the start of the study. Parent(s) completed a *Home Learning Environment Questionnaire* (Dulin et al., 2023) which characterised (a) HLE richness and (b) child engagement in SBR. Language skills were measured using a parent-completed vocabulary checklist and a standardised measure of expressive vocabulary at two time points approximately 9 months apart.

**Results/discussion:**

Descriptive statistics revealed variability in the richness of HLEs (*M* = 32.76, *SD* = 6.83, *range* = 16–44) and child engagement during SBR (*M* = 18.86, *SD* = 6.00, *range* = 5–32). Parents of children with Down syndrome reported rich and variable home literacy environments, and frequent shared book reading in which there were generally high levels of child engagement, with children reported to regularly request books, turn pages, and point to pictures or words. Child engagement during SBR was the strongest predictor of concurrent expressive vocabulary, suggesting immediate vocabulary growth is linked with child engagement. Although HLE richness was not a significant predictor of vocabulary at either time point, stronger correlations between HLE richness and vocabulary at Time 2 suggest possible cumulative or lagged effects as children’s language becomes more sensitive to environmental input over time.

## 1 Introduction

Recent research has increasingly focused on the cognitive and behavioural implications associated with neurogenetic conditions. Once such condition is Down syndrome (DS) (Fidler, 2005), which is the most common cause of learning disability, affecting 1 in every 737 births (Parker et al., 2010) and is caused by a trisomy of chromosome 21 (Jacobs et al., 1959; Lejeune et al., 1959; Silverman, 2007). DS is associated with mild to moderate intellectual disability and an uneven cognitive profile of strengths and weaknesses (Onnivello et al., 2022), including language delays, especially regarding expressive vocabulary and grammar (Abbeduto et al., 2007). Emergent language skills predict successive academic (e.g., literacy; Hulme et al., 2012), and psychosocial outcomes (Snowling et al., 2006) and the HLE plays an important role in typically developing (TD) children’s early language development (Lovčević, 2025). There has been paucity of research into the effects of the Home Literacy Environment (HLE) on the acquisition of language in children with DS (Dulin et al., 2023). The current study aims to characterise the HLEs of children with DS and the association between HLEs and vocabulary concurrently and longitudinally in children with DS.

Although the general trajectory of language acquisition for children with DS follows that of TD children (Næss et al., 2021), it is both delayed (Abbeduto et al., 2003) and, in some respects, different (Stoel-Gammon, 1980), with early milestones—such as canonical babbling (Lynch et al., 1995) and first-word production (Hoff, 2001)—emerging later than in TD children. Children with Down syndrome have a wide range of language abilities (Martin et al., 2009) and possess a distinct language profile characterised by relative delays across linguistic domains compared to their TD peers (Chapman and Hesketh, 2000). Delays in phonological awareness, verbal short-term memory, and speech intelligibility (Miller and Leddy, 1998) contribute to atypical language acquisition (Næss et al., 2011). Although speech intelligibility may improve with age (Roberts et al., 2007), language delays persist beyond what would be expected based on nonverbal mental age (Wild et al., 2018). While some studies suggest the development of syntax in children with DS aligns with that of nonverbal mental-age-matched typically developing (TD) peers (Miller et al., 1999), there is also evidence of more pronounced difficulties with syntax (Næss et al., 2011). Furthermore, pragmatic skills have been found to be impaired relative to chronological age matched TD peers (Smith et al., 2017). Additionally, up to 16–18% of children with DS have co-occurring Autism Spectrum Disorder (ASD) (Wang et al., 2025), which can further influence pragmatic language use.

Within this broader language profile, vocabulary acquisition is of particular importance, as single-word learning is a relative strength in children with DS compared to grammar (Abbeduto et al., 2007), and predicts later grammatical development (Miller et al., 1999). Expressive vocabulary is typically weaker in children with DS relative to their receptive vocabulary (Seager et al., 2022), though some studies suggest delays in both receptive and expressive vocabulary relative to TD peers matched on nonverbal mental age (Roberts et al., 2007). Parental reports on very young children (8–29 months) have found no significant differences in early expressive vocabulary between children with DS and TD peers (Galeote et al., 2011); however, naturalistic observations suggest more limited vocabulary in children with DS (Miller, 1995). Given that vocabulary underpins literacy and broader language development (Donolato et al., 2023), understanding these differences in receptive and expressive vocabulary is essential for identifying targets for language intervention.

These variations in language development align with the neuroconstructivist perspective, which views cognitive development as shaped by dynamic interactions between neuroanatomy, genetic factors, behaviour and the environment (Dekker and Karmiloff-Smith, 2011). Given this, the immediate environment of children with DS plays a crucial role in their cognitive development (Deckers et al., 2019). Family influences are particularly significant for children’s learning (Kaya et al., 2025), especially during childhood (Bornstein, 2002). Thus, enriching the home learning environment and particularly early language and literacy experiences taking place in the home (i.e., the Home Literacy Environment (HLE)) may potentially influence vocabulary growth in children with DS (Lusby and Heinz, 2020; Ranzato et al., 2021; Hilvert et al., 2023; Dulin et al., 2023).

### 1.1 Home literacy environments and language development

The HLE is a multifaceted construct that measures the quality and quantity of language-related experiences within the family home (DeTemple, 2001; Ranzato et al., 2021), independently and with others (e.g., family members; Justice et al., 2016). Since children with DS have variable cognitive profiles (Onnivello et al., 2022), the HLE can be tailored to provide different forms and levels of support in children’s language development (Burgoyne and Cain, 2022). Enriched HLEs have been shown to positively influence language and vocabulary growth in TD children (Bus et al., 1995; Liu and Li, 2022). However, fewer studies have examined its impact on children with DS (e.g., Lusby and Heinz, 2020; Burgoyne and Cain, 2022; Dulin et al., 2023). Existing evidence suggests that rich HLEs promote vocabulary growth in children with DS (Næss et al., 2021; Dulin et al., 2023). The “Home Literacy Model” (Sénéchal and LeFevre, 2014) suggests that the HLE consists of formal (attention is on the print material, e.g., explicitly teaching alphabet letters) and informal (print is present but not the focus of the parent–child interaction, e.g., SBR) literacy experiences. Informal literacy experiences are strongly associated with later language development in TD children (Sénéchal and LeFevre, 2014). The HLE is primarily parent-reported (Lusby and Heinz, 2020; Dulin et al., 2023), assessing the availability of print-rich activities, such as library trips and number of books available within the home (Peeters et al., 2009) and the frequency, duration and quality of parent–child shared book reading (SBR) interactions (Pillinger and Wood, 2014).

A large body of research with typically developing children demonstrates that sharing books with children can support oral language skills, particularly vocabulary development (Barone et al., 2019; Dowdall et al., 2020; Flack et al., 2018; Galea et al., 2025; Mol et al., 2008; Noble et al., 2020) though effect sizes vary depending on the design of the study (particularly the inclusion of an active control group; Noble et al., 2020) and the duration of intervention (Dowdall et al., 2020; Noble et al., 2020). Studies involving children with DS have also shown language promoting benefits of increased and sustained SBR on child language development (Jeremic et al., 2023). Evidence from diverse populations highlights the universal benefits of SBR on language development (Dowdall et al., 2020). For instance, Carmiol et al. (2024) found a significant positive association between SBR frequency and expressive vocabulary in a large Costa Rican sample (*n* = 183), where children had limited access to books. This study underscores the high value of SBR, even in low-resource settings, where there were minimal pre-existing exposure effects from SBR. Compared to non-reading activities, SBR facilitates richer language input (Gilkerson et al., 2017) by exposing children to a greater number of conversational turns, novel vocabulary and complex syntactic structures beyond everyday conversation (Pillinger and Wood, 2014). This is especially critical for children with DS, who benefit from language scaffolding and modelling (Seager et al., 2022). Within shared reading, children with DS rely on scaffolding more than TD children as children with DS initiate interaction less (Mattie and Fanta, 2023). Through SBR, parents can adjust their input through clarification, questioning and paraphrasing to meet their child’s needs (Hilvert et al., 2023), and encourage physical engagement (e.g., turning pages, pointing to pictures) and phonetic mimicry (e.g., requesting imitation) (Haden et al., 1996) to foster language development in children with DS (Barnes and Puccioni, 2017). SBR is a key feature of enhancing language development (Zuckerman et al., 2019) in the HLE, with both SBR frequency and quality being positively associated with children’s grammatical complexity (Hilvert et al., 2023) and improvements in receptive (Dulin et al., 2023) and expressive (Næss et al., 2021) vocabulary.

### 1.2 Home literacy environment and Down syndrome

Research on the HLE of children with DS presents a mixed picture (Trenholm and Mirenda, 2006; Ricci, 2011; Ranzato et al., 2021; Muscat and Grech, 2025). Several studies describe relatively rich HLEs, characterised by frequent book exposure and interactive shared reading (Al Otaiba et al., 2009; van Bysterveldt et al., 2010; Westerveld and van Bysterveldt, 2017; Lusby and Heinz, 2020; Næss et al., 2021; Burgoyne and Cain, 2022; Dulin et al., 2023). For example, Burgoyne and Cain (2022) found that caregivers of young children with DS (*n* = 8; aged 4–6 years) engaged in SBR daily or multiple times per day. Similarly, Dulin et al.(2023) found that all mothers of toddlers with DS aged 11–29 months (*n* = 13) introduced reading before their child’s first birthday and had many child-level books (*M* = 122.31, *SD* = 99.26, *range* = 30–300) and adult-level books (*M* = 120.77, *SD* = 74.77, *range* = 20–200) at home. The majority also reported using interactive reading strategies, such as pointing out details beyond the written text (*n* = 13/13) and relating the story to the child’s experiences (*n* = 10/13).

However, other research indicates that frequency of literacy activities may be lower in some families of children with DS, despite the presence of books in the home (Muscat and Grech, 2025). Trenholm and Mirenda (2006), in a large caregiver survey (*n* = 224), found that most caregivers did not read daily and often limited SBR to brief sessions, with only a quarter using interactive techniques. Studies including comparison groups similarly suggest that HLEs may be less rich than those of typically developing children (Ricci, 2011; Ranzato et al., 2021; Næss et al., 2021; Muscat and Grech, 2025). For instance, Ricci (2011) reported that preschoolers with DS were exposed to less print-rich materials than both school-aged children with DS and age-matched TD peers, while Muscat and Grech (2025) observed higher daily reading rates among families of TD children (47.6%) than families of children with DS (45%).

These discrepancies likely reflect the influence of child factors, parental characteristics, culture and socio-economic status (SES) (Daniels et al., 2022). Most existing DS HLE research has focused on middle- to high-SES families (Al Otaiba et al., 2009; Burgoyne and Cain, 2022; Dulin et al., 2023), limiting generalisability. SES clearly shapes literacy practices: lower-SES families of TD children engage in fewer literacy activities (Newman, 1996; Rosslund et al., 2025), For instance, Muscat and Grech (2025) compared the HLEs of TD children and children with DS and found that, across the groups, only 40% of parents from low-SES families read to their children daily, compared to 70% of middle-SES and 80% of high-SES parents. Moreover, HLEs and SES in children with DS are largely influenced by family background and literacy opportunities, which are largely context-specific (Inoue et al., 2020). For instance, a study conducted in Malta found that higher parental education was associated with richer HLEs, including more books, supportive literacy practices, and positive parental attitudes toward reading (Muscat and Grech, 2025). Similarly, surveys in Canada and the UK indicate that many families provide a variety of reading materials and engage in literacy activities with their children, though actual use by children varies and may be influenced by parental resources and motivation (Trenholm and Mirenda, 2006). In New Zealand, parents reported active literacy engagement with children with DS, though SES was not systematically measured (van Bysterveldt et al., 2010). Across these studies, higher parental education or proxy SES measures generally correspond to richer HLEs, highlighting the role of socio-economic disparities. However, no multi-country studies exist, limiting cross-cultural generalisations and underscoring the need for harmonized international research on SES, HLE, and literacy in DS populations.

Although the HLE plays a crucial role in language development among TD children (Galea et al., 2025), some research suggests that its direct impact on language acquisition in children with DS may be more limited. Instead, interest in reading appears to be more strongly related to language outcomes for children with developmental disabilities (Justice et al., 2016), and the frequency of SBR has been shown to correlate more reliably with increased reading interest than with direct language gains (Ricci, 2011). In TD populations, informal literacy experiences, such as SBR, are more strongly associated with language development, whereas formal literacy experiences tend to predict reading skills more specifically (Sénéchal and LeFevre, 2014). It remains unclear, however, whether this distinction holds for children with DS, whose language profiles and learning trajectories differ from those of TD children. Consequently, the relationship between the HLE and language development in DS may depend less on the frequency of literacy activities alone and more on children’s level of engagement and participation during SBR interactions (Wang et al., 2022). High engagement during SBR is particularly beneficial for children with DS (Abbeduto et al., 2007), as they may require additional scaffolding to support their language development. Despite this, most questionnaires measuring the HLEs of children with DS focus predominantly on caregiver behaviours, with few studies including questions measuring child engagement (van Bysterveldt et al., 2010; Westerveld and van Bysterveldt, 2017; Dulin et al., 2023). Existing research suggests that children with DS are highly engaged during SBR (Lusby and Heinz, 2020), although some studies suggest children with DS may take a more passive role during reading activities when compared to their developmental age-matched peers (van Heerden and Kritzinger, 2008; Al Otaiba et al., 2009).

Lusby and Heinz (2020) found that 70% of caregivers of children with DS (1-to 6-year-olds) reported their child ‘often/always’ points to and/or names pictures and 50% of caregivers reporting children ‘often/always’ name pictures during SBR. Although only 33% of children ‘often/always’ commented on the story. Similarly, van Bysterveldt et al. (2010) assessed more complex engagement behaviours in school-aged children with DS (e.g., asking questions) and found lower engagement, with 45% of children ‘often/ usually’ commenting on pictures and only 10–30% of children asking questions about story features. Ricci (2011) found that school-aged children with DS were exposed to richer HLEs and had greater engagement during SBR than pre-school-aged children with DS. However, whilst these studies suggest engagement behaviours differ by age group, it is unclear whether the lower levels of engagement observed in school-aged children (van Bysterveldt et al., 2010) reflect genuine age-related differences, or whether the behaviours assessed (e.g., asking questions) were too complex for the children’s developmental level, thus underestimating their true engagement. Nevertheless, findings suggest that HLE predictors, particularly engagement, may be age dependent.

### 1.3 The importance of child engagement in shared book reading and language development

Child engagement during SBR plays a crucial role in language development, potentially outweighing the direct influence of HLE richness. Dulin et al. (2023) found that mothers of children with DS (*n* = 13; 11–29 months of age) reported rich HLEs and high child engagement in SBR, with child engagement significantly correlating with both concurrent (*r* = 0.82, *p* < 0.001) and longitudinal measures of receptive vocabulary taken 6 months later (*r* = 0.89, *p* < 0.001). However, the authors did not find a significant association between HLE richness and vocabulary scores concurrently or longitudinally (*r* = 0.41 and *r* = 0.52 respectively). Although small sample sizes are common with this population, they limit our ability to fully comprehend the impact individual differences have on the results, so may inflate the risk of errors (Button et al., 2013). Moreover, these authors (Dulin et al., 2023) focused on young toddlers, so the authors were unable to explore associations between the HLE and expressive vocabulary, which could be observed in older children with DS who have more advanced language (Ricci, 2011). Given the variable receptive and expressive vocabulary among children with DS (Roberts et al., 2007), assessing these different subsystems separately would enable an understanding of their specific associations with HLE characteristics. Additionally, the study relied on parent-reported language measures, which while offering valuable insights, are subject to potential biases and recall limitations (Feldman et al., 2000).

In summary, existing evidence suggests children with DS have variable HLEs (Trenholm and Mirenda, 2006) and that child engagement during SBR may be positively correlated with receptive language skills (Dulin et al., 2023). However, the extent to which the HLE influences expressive language development in children with DS remains unclear (Dulin et al., 2023). Furthermore, few longitudinal studies have investigated the role of the HLE in vocabulary growth for children with DS, with most being small-scale (*n* = 13; Dulin et al., 2023). Consequently, there remains limited knowledge on how the HLE shapes vocabulary development for children with DS—an area critical for informing targeted language-enhancing interventions (Jeremic et al., 2023).

The current study examined the relationship between the HLE and child vocabulary, in sample of 29 children (ages 2–6 years) with DS, addressing the following research questions:

1. How do parent(s) of children with DS describe their child’s HLEs?
2. How do parent(s) of children with DS describe their child’s engagement throughout SBR?
3. Do measures of HLE richness and child engagement correlate with concurrent measures of expressive and receptive vocabulary?
4. Do measures of HLE richness and child engagement predict expressive and receptive vocabulary longitudinally?

Based on prior research, we hypothesised that richer HLEs and greater child engagement in SBR would be associated with higher concurrent and longitudinal expressive and receptive vocabulary scores.

## 2 Materials and methods

### 2.1 Participants

The data used in this study was collected as part of a feasibility randomised controlled trial of an early language intervention for children with DS (Burgoyne et al., 2023). For this trial, thirty children with DS aged 3–6 years and their parents, living within 40 miles of study sites in the North and South regions of England (University of Manchester/University of Reading) were recruited via UK Down syndrome organizations, regional Down syndrome support groups in England, Regional Hubs and the Down syndrome Clinical Excellence Network of the Royal College Speech and Language Therapists and regional Educational Psychology services as well as social media. Children had to have a minimum cognitive level of 18 months assessed by a trained researcher using the Visual Reception and Fine Motor subscales of the Mullen Scale of Early Learning (Mullen, 1995) and a minimum expressive vocabulary of 10 (English) words/signs assessed online using the parent-report Reading Communicative Development Inventory (CDI) (Hamilton et al., 2016). Twins were excluded from the study. Parents had to read and speak English to take part.

The data reported in the current study were collected in baseline assessments (i.e., conducted prior to group allocation and intervention) with 29 parent–child dyads from the main study: One dyad from the original 30 did not complete the HLE questionnaire and was not therefore included in this study. Participating children (66% male/34% female) were aged 2 years and 11 months—6 years (age 2–3 years: *n* = 1; 3–4 years: *n* = 13; 4–5 years: *n* = 4; 5–6 years: *n* = 7; and 6–7 years: *n* = 4). Twelve of these families also provided data 9 months after the initial data collection (Time 2). These families were those randomly allocated to a control group in the main study who did not receive intervention between Time 1 and Time 2 (*n* = 12; children aged 3 years and 9 months—7 years and 8 months).

Parents (both parents unless children were living in single-parent households) were asked to report their current occupations and highest formal qualification level. Parental occupations were coded according to the Standard Occupational Classification (Socioeconomic Classification, 2020). In instances where parents were currently not employed, their previous occupation was recorded. Fifty-seven parents submitted responses (95% of all parents). Most parents were in Professional (63%, *n* = 36) or Associate Professional roles (16%, *n* = 9), with smaller numbers of parents in Manager, Director and Senior Official roles (7%, *n* = 4), Administrative and Secretarial roles (4%, *n* = 2), Skilled Trades (4%, *n* = 2) or Caring, Leisure and Other Service roles (7%, *n* = 4). Fifty-six parents reported highest qualification levels (93%). Most reported having a degree at a postgraduate (48%, *n* = 27) or undergraduate level (30%, *n* = 17). A further 9% of parents (*n* = 5) had a vocational qualification, 7% (*n* = 4) reported their highest level of education as A-levels, and 5% (*n* = 3) as General Certificate of Secondary Education (GCSEs). No parents indicated they had ‘No Formal Qualifications’.

### 2.2 Procedures

Assessments were completed at the start of the study (Time 1); language assessments were completed for a second time approximately 9 months later (Time 2). In-person assessments were conducted by a trained researcher on campus; in a small number of cases, assessments were completed at the family’s home or the child’s nursery/school to enable testing in a familiar setting to support child engagement or accommodate parents unable to travel to the University site.

### 2.3 Measures

#### 2.3.1 Home-literacy environment (HLE) questionnaire

A parent-completed online HLE questionnaire (Dulin et al., 2023) was used to characterise the richness of the HLE and child engagement during SBR activities. The questionnaire contained Likert-type (*n* = 6), forced-choice (*n* = 19) and short-answer (*n* = 3) questions. The questionnaire contained one question pertaining to formal literacy activities (teaching letters), and the rest asked about informal literacy activities (e.g., frequency of SBR). Two composite variables were calculated from responses to characterise the HLE: (1) parent-reported HLE richness and (2) parent-reported child engagement during SBR. Items and scoring information can be found in Appendix A.

##### 2.3.1.1 Richness of HLE

The HLE richness composite (Cronbach’s alpha = 0.64) contained 13 questions, which measured the quality and quantity of literacy-related activities within the home environment. Items measured the accessibility to adult- and children-level books, the duration and frequency of SBR with their child, and the use of interactive reading strategies. Each response was given a corresponding value (see Appendix A), and the sum of these items was calculated into a total score, with higher scores indicating a richer HLE (highest possible score: 67).

##### 2.3.1.2 Child engagement

The child engagement composite (Cronbach’s alpha = 0.70) contained eight questions measuring child behaviour in a typical SBR interaction with their parent. The response on each item was given a corresponding value (see Appendix A), and the sum of these eight items was calculated into a total score. This total score indicated child engagement in SBR, with higher scores suggesting greater child engagement during shared reading interactions (highest possible score: 36).

#### 2.3.2 Vocabulary

Children’s vocabulary was measured at Times 1 and 2 using the parent-reported Reading Communicative Development Inventory (CDI) (Hamilton et al., 2016). This measure was derived from the MacArthur-Bates Communicative Development Inventory (Fenson et al., 2007), which has strong reliability and validity (i.e., internal consistency = 0.95–0.96, test–retest reliability = 0.61–0.85, correlates strongly with the Bayley Scales of Infant Development = 0.77 and the EOWPVT = 0.73–0.85; Fenson, 2007; Heilmann et al., 2005; Dale, 1991). However, unlike the original CDI, it measures both spoken and signed vocabulary (understanding and use), making it particularly useful for children with DS, who often sign at early ages. Test–retest reliability coefficients for the CDI range from 0.70 to 0.75 (Duff et al., 2015), and it has been successfully used for participants with DS in previous studies (Mason-Apps et al., 2018). The raw score of ‘words understood’ variable was used as a measure of receptive vocabulary and the raw score ‘words produced’ variable was used as a measure of expressive vocabulary.

A trained examiner administered the EOWPVT (Brownell, 2010), a picture-based test assessing single-word naming of everyday actions, objects, and concepts from pictures. The EOWPVT (for ages 2 to 80+) demonstrates high internal consistency, test–retest reliability, and interrater reliability, with coefficients ranging from 0.93 to 0.98 (*median* = 0.98) (Gardner, 1981). It also has moderate to high correlations (0.41 to 0.80) with established language measures, such as the *Peabody Picture Vocabulary Test* (PPVT-R; Dunn and Dunn, 1965) and *the Wechsler Intelligence Scale for Children—Revised* (WISC-R-VIQ) (Wechsler, 1974), supporting its concurrent and construct validity for testing vocabularies of children with learning difficulties (Furlong and Teuber, 1984). The total raw score was used as a measure of vocabulary.

#### 2.3.3 Non-verbal IQ (NVIQ)

NVIQ was measured to screen children against inclusion criteria (minimum NVIQ of 18 months) and in this study is included as a control variable. A trained examiner measured children’s general cognitive abilities at Time 1 using the *Mullen Scales of Early Learning* (MSEL) (Mullen, 1995). Averaged age equivalent scores on the Visual Reception and Fine Motor subsets were calculated as a measure of nonverbal IQ (NVIQ). The MSEL has accepted content and construct validity (Mullen, 1995) and is concurrently valid alongside similar standardised tests of early child development of cognition (e.g., the *Peabody Developmental Motor Scales* Folio and Fewell, 1983) and *Bayley Scales of Infant Development* (Bayley, 1993). Moreover, it holds high test–retest reliability (0.82 to 0.85), interrater reliability (0.91 to 0.99) and internal consistency (0.83 to 0.95) (Mullen, 1995).

### 2.4 Data analysis plan

Preliminary analyses revealed no violation of normality, linearity, multicollinearity or homoscedasticity assumptions. Tests for normality were assessed by observing visual plots (Q-Q plot) and conducting Shapiro–Wilk test for normality, which is widely regarded as one of the most powerful tests of normality and is particularly suitable for small to moderate sample sizes (Shapiro and Wilk, 1965; Razali and Wah, 2011). To address research questions 1 and 2, descriptive statistics were used to summarise how the parents of children with DS reported their HLEs and their child’s engagement during SBR. For research questions 3 and 4, Pearson’s *r* correlations were conducted to examine the associations between predictor variables and children’s vocabulary scores. Multiple linear regression was then performed to examine if the richness of parent-reported HLE, or parent-reported child engagement during SBR interactions, was associated with vocabulary scores at Time 1 and predicted vocabulary scores at Time 2. Child engagement composite and HLE composite scores were used as predictors. Outcome variables were receptive and expressive vocabulary scores. In significant models, age was added as a control variable in follow-up analyses (note that regression models where NVIQ was controlled for in place of age were also run, but these are not reported here as the pattern of results stayed the same). Analyses were run on R Studio software (RStudio Team, 2023). Regression models were fitted using the base R stats package (lm() function). Model fit was assessed using goodness-of-fit metrics (R-squared, adjusted R-squared, and residual standard error), significance of predictors (*p*-values for coefficients and F-statistics from the model summary), assumption checks (residual diagnostics were visually inspected using standard diagnostic plots (plot() function on the fitted model) to assess linearity, normality, homoscedasticity, and influential points), as well as additional tests (such as the Shapiro–Wilk test for normality (shapiro.test()), Breusch-Pagan test for heteroscedasticity (bptest() from the lmtest package), and variance inflation factors (vif() from the car package) were used to check model assumptions) where appropriate. Regression models were compared using the Akaike Information Criterion (AIC) and ANOVAs.

## 3 Results

### 3.1 Research question 1: how do parents of children with DS describe their HLEs?

All parents reported they started reading to their child between birth and 12 months of age (*M* = 3.28 months, *SD* = 3.36 months). Parents reported having a wide range of child-level books (*n* = 27, *M* = 97.41, *SD* = 66.99, *range* = 20–300) and adult-level books (*n* = 27, *M* = 178.70, *SD* = 287.47, *range* = 10–1,200) within the family home. Most parents read to their child for 1–2 h (*n* = 16) or 3–4 h (*n* = 9) per week, or 10–20 min per sitting (*n* = 15). Moreover, they reported using various interactive strategies during a typical SBR session with their child (*n* = 26 point out additional story details not within the written text; *n* = 20 relate the story to their child’s everyday actions; *n* = 18 ask their child questions about the story and follow up with answers). Fewer parents (*n* = 13) reported teaching names or sounds of alphabet letters during SBR. Further frequency responses for additional HLE items can be found in Table 1.

**Table 1:** The frequency of parents’ responses on items on the HLE questionnaire related to the richness of the HLE.

Question	Never/0	1–2	3–4	5–6	7–8	9–10	11+
How many times in a week parent reads books for enjoyment	10	8	4	3	3	1	0
How many times in a week parent reads informative books	8	9	5	2	3	0	2
How many times in a week parent reads to child	1	1	5	3	7	2	10
How many books parent read to child in past week	0	3	5	6	4	2	9
How many books parent reads to child in one sitting	0	22	7	0	0	0	0
	**<15 min**	**15–30 min**	**30–45 min**	**1–2 h**	**3–4 h**	**5–6 h**	**7 + h**
How much time parent spent reading to child in past week	1	1	2	16	9	0	0
	**<10 min**	**10–20 min**	**21–30 min**	**31–40 min**	**41–50 min**	**51–60 min**	**>1 h**
How much time parent reads to child in one sitting	11	15	3	0	0	0	0
	**Never**	**Once**	**Every 4–6 mos**	**Every 2–3 mos**	**Monthly**	**Every 2–3 wks**	**Weekly**
How often parent took child to bookstore/library in the last year	4	3	7	5	5	5	0

Interactive book reading styles	Strongly disagree	Disagree	Agree	Strongly agree
Points out story details outside the actual text	0	3	12	14
Relates the story to child’s everyday actions	1	8	11	9
Ask questions and follows-up with answers	4	9	14	4
Teaches child letters/sounds of letters	7	9	9	4

*N* = 29. Min, Minutes; H(s), Hours(s); Wks, Weeks; Mos, Months.

### 3.2 Research question 2: how do parents of children with DS describe their child’s engagement during shared book-reading interactions?

Over half of parents reported that their child requests to be read to (*n* = 26) or pretends to read a book (*n* = 22) weekly. When reporting their child’s interactions during SBR, most parents reported that (‘Occasionally’, ‘A few times per story’, or ‘Very frequently’) their child grabs for/ holds the book (*n* = 27), turns the pages with help (*n* = 28), and independently points to words or pictures (*n* = 28). Fewer parents reported that on numerous occasions their child fills in words/ lines of a familiar story (*n* = 11). However, most parents reported that their child did not ask questions about the story (*n* = 21). Parents’ responses to the HLE questionnaire questions relating to child engagement can be found in Table 2.

**Table 2:** The frequency of parents’ responses on items on HLE questionnaire related to the child’s engagement.

Question	Never/0	1–2	3–4	5–6	7–8	9–10	11+
How many times child asks parent to read to them in a week	3	5	6	3	8	0	4
How many times child pretends to read books in a week	7	4	7	5	2	0	4

“During storybook reading time with your child, do they…”	Never	Has but rarely	Occasionally	A few times per story	Very frequently during story
Grab for/holds book	0	2	6	6	15
Turns pages of book with help	0	1	1	11	16
Independently points to pictures/words on page	0	1	13	6	9
Names familiar pictures	2	2	11	6	8
Asks questions about story	21	4	2	1	1
Fills in words/ lines of familiar story	10	8	6	2	3

*N* = 29.

### 3.3 Research question 3: how do HLE and child engagement relate to concurrent measures of vocabulary?

Descriptive statistics for each variable can be found in Table 3. There is considerable variability across all measures showing a wide range of individual differences in the sample. The results suggest children had variable levels of HLE richness (*M* = 32.76, *SD* = 6.83) and child engagement (*M* = 18.86, *SD* = 6.00). On average, scores on the vocabulary measures were higher at Time 2 than at Time 1 showing growth in vocabulary over time. On average, children had a higher receptive vocabulary, relative to expressive vocabulary (CDI) across both time points.

**Table 3:** Descriptive statistics for each variable.

Variable	M	S.D.	Range	N
Time 1 Chronological age, child^a^	54.90	13.97	35–82	29
Time 2 Chronological age, child^b^	63.31	24.87	45–92	12
NVIQ^c^	27.66	7.43	18–49	29
Richness HLE	32.76	6.83	16–44	29
Engagement HLE	18.86	6.00	5–32	29
CDI receptive				
Time 1	326.76	139.39	63–595	29
Time 2	421.18	129.76	218–594	11
CDI expressive				
Time 1	172.07	105.18	22–454	29
Time 2	267.36	154.00	82–495	11
EOWPVT				
Time 1	14.56	10.25	0–33	27
Time 2	26.00	12.13	7–45	12

a Child’s age at Time 1, reported in months.

b Child’s age at Time 2, reported in months.

c NVIQ at Time 1, Nonverbal IQ derived from Mullen Scales of Early Learning (MSEL).

Correlations among key variables at Time 1 can be found in Table 4. There was a significant moderate correlation between age and NVIQ (*r* = 0.48). The correlation between the HLE measures (richness and engagement) was relatively weak (*r* = 0.23) and was not significant. There were significant moderate to large correlations between age and vocabulary (CDI receptive *r* = 0.48; CDI expressive *r* = 0.39; EOWPVT *r* = 0.64) at Time 1. In addition, child engagement was significantly moderately correlated with CDI expressive (*r* = 0.53) and EOWPVT (*r* = 0.45) scores and there was a moderate correlation (*r* = 0.37) which approached significance with CDI receptive scores. There were no significant correlations between HLE richness and vocabulary scores.

**Table 4:** Correlations among predictors at Time 1 and language outcomes at Time 1.

Variable	1	2	3	4	5	6	7
1 Age	1.00	0.48***	0.02	0.11	0.48**	0.39*	0.64***
2 NVIQ	0.48**	1.00	0.01	0.20	0.32	0.16	0.61***
3 Richness			1.00	0.23	0.24	0.04	−0.08
4 Engagement				0.1.00	0.37†	0.53**	0.45*
5 CDI Receptive					1.00	0.76***	0.74***
6 CDI Expressive						1.00	0.75***
7 EOWPVT							1.00

^†^*p* = 0.051, * < 0.05, ** < 0.01, *** < 0.001. *N* = 29. Age, Age taken at Time 1; NVIQ, Nonverbal IQ derived from the Mullen Scales of Early Learning (MSEL); CDI receptive, Words Understood Score on the CDI; CDI expressive; Words Produced Score on the CDI.

Multiple linear regression models were conducted to explore the association between HLE richness and child engagement (see Table 5) and vocabulary measures at Time 1. The model predicting CDI receptive vocabulary was not significant, *F*(2, 26) = 2.46, *p* = 0.105, accounting for 16% of the variance in receptive vocabulary. The model predicting CDI expressive vocabulary was significant, *F*(2, 26) = 5.35, *p* = 0.011. This model accounted for 29% of the variance in expressive vocabulary, explained uniquely by child engagement. The model predicting EOWPVT vocabulary scores was also significant, *F*(2, 24) = 3.48, *p* = 0.047, accounting for 23% of the variance, with 21% unique variance from child engagement. There were no significant, unique effects from HLE richness in either of these models.

**Table 5:** Multiple linear regression predicting language measure outcomes at Time 1.

Language outcome	CDI receptive			CDI expressive			EOWPVT		
Predictor variable	Beta	*t*	*p*	Beta	*t*	*p*	Beta	*t*	*p*
HLE	3.38	0.90	0.370	−1.25	−0.48	0.636	−0.23	−0.79	0.439
Child engagement	7.62	1.78	0.088	9.69	3.26	0.003	0.81	2.61	0.016

*N* = 29.

Follow-up analyses examining the contribution of age to the overall models were conducted (see Table 6). Age was a significant predictor in all models (CDI receptive: *F*(1, 27) = 8.12, *p* = 0.008; CDI expressive: *F*(1, 27) = 4.77, *p* = 0.038; EOWPVT: *F*(1, 25) = 16.91, *p* < 0.001) accounting for 15 to 40% of the variance in vocabulary. Child engagement remained a significant predictor of CDI expressive vocabulary predicting 27% of the unique variance in CDI expressive vocabulary over and above age, which predicted 13% unique variance. Child engagement also remained a significant predictor of EOWPVT expressive vocabulary, predicting 20% of the unique variance in EOWPVT, and age predicted 39% of the unique variance in the model. Age was the only significant unique predictor in the CDI receptive model.

**Table 6:** Multiple linear regression predicting language measure outcomes at Time 1 accounting for age at Time 1.

Language outcome	CDI receptive				CDI expressive				EOWPVT			
Predictor variable	*B*	Beta	*t*	*p*	*B*	Beta	*t*	*p*	*B*	Beta	*t*	*p*
Model 1												
Age	0.48	5.04	2.85	0.008	0.39	3.07	2.18	0.038	−0.64	0.50	4.11	< 0.001
Model 2												
Age	0.45	4.67	2.76	0.011	0.33	2.62	2.12	0.044	0.61	0.48	4.56	< 0.001
HLE	0.17	3.40	1.01	0.322	−0.08	−1.24	−0.51	0.617	−0.06	−0.09	−0.42	0.679
Child engagement	0.28	6.44	1.67	0.107	0.51	9.03	3.22	0.004	0.44	0.75	3.23	0.003

*N* = 29. CDI Receptive: R^2^ = 0.23 for Model 1; R^2^ change = 0.13 for Model 2; total R^2^ = 0.36. CDI Expressive: R^2^ = 0.15 for Model 1; R^2^ change = 0.25 for Model 2; total R^2^ = 0.40. EOWPVT: R^2^ = 0.40 for Model 1; R^2^ change = 0.19 for Model 2; total R^2^ = 0.59. HLE, Richness of HLE.

### 3.4 Research question 4: do measures of the HLE (richness and child engagement) relate to vocabulary outcomes longitudinally?

Correlations between predictors at Time 1 and vocabulary measures at Time 2 can be found in Table 7. Age was moderately correlated with all vocabulary measures, but the correlation was significant only for EOWPVT (*r* = 0.64). Child engagement was significantly correlated with CDI receptive (*r* = 0.62) and EOWPVT (*r* = 0.64) scores at Time 2; though there was a moderate correlation with CDI expressive scores (*r* = 0.56) this was not significant. Weaker correlations were found between HLE richness and vocabulary, and these were not significant.

**Table 7:** Correlations among predictors at Time 1 and language outcomes at Time 2.

Variable	1	2	3	4	5	6	7
1 Age	1.00	0.48**	0.02	0.26	0.53	0.57	0.64*
2 NVIQ		1.00	−0.08	0.46	0.14	−0.04	0.47
3 Richness			1.00	0.23	0.37	0.37	0.21
4 Engagement				0.1.00	0.62*	0.56	0.64*
5 CDI Receptive					1.00	0.91***	82**
6 CDI Expressive						1.00	0.75**
7 EOWPVT							1.00

* < 0.05, ** < 0.01, *** < 0.001. *N* = 12. Age, Age taken at Time 1; NVIQ, Nonverbal IQ derived from the Mullen Scales of Early Learning (MSEL); CDI receptive, Words Understood Score on the CDI; CDI expressive; Words Produced Score on the CDI.

A series of multiple linear regression models were conducted, predicting each of the vocabulary measures at Time 2 from the HLE composites of richness and child engagement (see Table 8) at Time 1. The models predicting CDI receptive scores, *F*(2, 8) = 2.55, *p* = 0.139, accounting for 39% of the variance, and CDI expressive scores, *F*(2, 8) = 1.92, *p* = 0.208, accounting for 32% of the variance, were not significant. The model predicting EOWPVT scores was non-significant, *F*(2, 9) = 3.72, *p* = 0.067, accounting for 45% of the variance in EOWPVT scores; however, examination of the predictor variables showed significant, unique effects of child engagement explaining 41% unique variance (*p* = 0.030). There was no significant, unique contribution from HLE richness.

**Table 8:** Multiple linear regression predicting vocabulary at Time 2.

Language outcome	CDI receptive			CDI expressive			EOWPVT		
Predictor variable	Beta	*t*	*p*	Beta	*t*	*p*	Beta	*t*	*p*
HLE	1.57	0.27	0.793	2.76	0.38	0.712	−0.41	−0.84	0.424
Child engagement	12.11	1.81	0.108	12.34	1.48	0.117	1.46	2.58	0.030

*N* = 12.

Follow-up analyses examining the contribution of age at Time 1 on vocabulary measures at Time 2 were conducted for the models (see Table 9). None of the models were significant overall. Age was a significant predictor for EOWPVT, *F*(1, 10) = 7.20, *p* = 0.023, accounting for 42% of the variance in EOWPVT scores. The effect of child engagement after controlling for age was not significant (*p* = 0.054). HLE and child engagement were not significant predictors in any models.

**Table 9:** Multiple linear regression predicting language measure outcomes at Time 2 accounting for age at Time 1.

Language outcome	CDI receptive				CDI expressive				EOWPVT			
Predictor variable	*B*	Beta	*t*	*p*	*B*	Beta	*t*	*p*	*B*	Beta	*t*	*p*
Model 1												
Age	0.53	4.35	1.87	0.094	0.57	5.52	2.06	0.069	0.65	0.47	2.68	0.023
Model 2												
Age	0.26	2.17	0.76	0.470	0.37	3.63	1.06	0.325	0.49	0.36	2.30	0.050
HLE	0.09	1.71	0.29	0.781	0.14	3.00	0.42	0.688	−0.15	−0.24	−0.59	0.572
Child engagement	0.42	8.86	1.10	0.309	0.28	6.92	0.71	0.500	0.60	1.11	2.26	0.054

*N* = 12. CDI Receptive: R^2^ = 0.28 for Model 1; R^2^ change = 0.16 for Model 2; total R^2^ = 0.44. CDI Expressive: R^2^ = 0.32 for Model 1; R^2^ change = 0.10 for Model 2; total R^2^ = 0.42. EOWPVT: R^2^ = 0.42 for Model 1; R^2^ change = 0.25 for Model 2; total R^2^ = 0.67. HLE, Richness of HLE.

## 4 Discussion

The current study examines the HLEs of children with DS and their association with vocabulary development, both concurrently and longitudinally. Data was collected to describe the HLEs of children with DS, their engagement during SBR interactions at home, to analyse how these factors relate to vocabulary development. Although the HLE is a well-established predictor of language development in TD children (Galea et al., 2025), research on this relationship in children with DS remains limited (Dulin et al., 2023).

The findings from the current study suggest that 3-6-year-old children with DS, from moderate to high SES families, have variable HLEs and child engagement. Variations in child engagement were moderately correlated with measures of expressive and receptive vocabulary assessed concurrently (*r’s* = 0.37–0.53) and longitudinally (*r’s* = 0.56–0.64). Child engagement was a significant predictor of expressive vocabulary (but not receptive vocabulary) at Time 1, and predicted scores on the EOWPVT measures of expressive vocabulary at Time 2, though this was no longer significant when controlling for age (*p* = 0.054). HLE richness was not a significant predictor of vocabulary at either time point. The following sections discuss these findings and their implications.

### 4.1 The HLE of children with DS

The first two research questions aimed to characterise how parents of children with DS report their HLEs and SBR interactions. Parents indicated that their homes contained, on average, 97 children’s books. In the current study, parent–child reading frequency varied widely, from ‘Never/0 times’ (3%, *n* = 1) to ‘11 + times’ (34%, *n* = 10) per week, with over half of parents reporting reading to their child 7–11 times per week and most reading sessions lasting 10–20 min. Library or bookstore visits showed similar variability, ranging from ‘Never’ (14%, *n* = 4) to ‘Every 2-3 weeks’ (17%, *n* = 5) in the past year. These findings align with previous literature and suggest that children with DS experience variable HLEs including access to books and frequency of SBR interactions (e.g., Ranzato et al., 2021; Burgoyne and Cain, 2022), which has been suggested to be possibly influenced by their cognitive profiles and learning needs (Ranzato et al., 2021). Most parents also reported using interactive reading styles during SBR, which is consistent with previous research. For instance, Barton-Hulsey et al. (2020) observed that mothers of children with DS tend to ask more questions and use more gestures, labels and descriptions during SBR interactions compared to mothers of TD children.

Parents also reported their child’s engagement during SBR interactions. Most parents reported that, in a typical week, their child requests to be read to or pretends to read a book independently. Although the frequency of specific engagement behaviours varied, many parents reported that their child often grabs or holds books, turns pages with help, and independently points to pictures or words. The engagement questions were those used by Dulin et al. (2023), who examined engagement patterns in toddlers (*n* = 13; 11–29 months of age). Our study found higher levels of child engagement (*M* = 18.86, *SD* = 6.00) compared to those reported for toddlers by Dulin *et al.* (*M* = 12.38, *SD* = 6.38). These results align with previous observations that engagement in SBR increases with age in children with DS (Ricci, 2011).

It is important to consider whether the questionnaire items used to assess engagement may tap behaviours that are more developmentally accessible to older children (Sénéchal and LeFevre, 2014). Many of the items, such as independently turning pages, pointing to pictures, or pretending to read, require motor, attentional, and symbolic skills that typically emerge later in development (Roberts et al., 2007). Thus, it may not be surprising that these behaviours are reported more frequently in older samples. The higher engagement scores observed in the present study, relative to the toddler sample by Dulin et al. (2023), may therefore reflect not only genuine differences in engagement but also age-related differences in children’s capacity to perform these behaviours.

Children who are more engaged in SBR are more likely to experience opportunities to develop their expressive vocabulary (Hilvert et al., 2023) and receive more opportunities for responsive language input from caregivers (Mattie and Fanta, 2023). Overall, these findings suggest that children with DS in our sample are engaged during SBR, and that the developmental appropriateness of the engagement behaviours measured through the questionnaire may partly account for higher engagement levels observed in older cohorts, relative to previous research (Dulin et al., 2023).

### 4.2 Predicting child language development

The third and fourth research questions examined whether HLE richness and child engagement were related to children’s vocabulary concurrently and 9 months later. Child engagement emerged as the strongest, unique predictor of concurrent expressive vocabulary and this remained significant after controlling for age. Children with higher engagement may spend more time in SBR (van der Schuit et al., 2009), receiving greater scaffolding from caregivers (Lusby and Heinz, 2020), thus deriving more linguistic benefits from the interactions. This relation may also be bidirectional, as children with stronger vocabularies may enjoy SBR more, increasing engagement (Justice et al., 2016). Thus, these children may be more likely to spend greater time SBR with their caregiver, facilitating stronger vocabulary development (Mattie and Hadley, 2021). The link between SBR and language development is well-documented (e.g., Burgoyne and Cain, 2022), though effect sizes vary widely across studies (Jeremic et al., 2023).

Neither HLE measures (HLE richness or child engagement) predicted receptive vocabulary. This may be because children with DS have stronger receptive vocabularies relative to their expressive vocabularies (Chapman and Hesketh, 2000). Since expressive language is disproportionately impaired relative to receptive language in children with DS, it may be more sensitive to variation in environmental input (such as high-quality SBR; Naigles and Tek, 2017). Moreover, because receptive vocabulary is relatively stronger in children with DS, it may show less measurable variation in response to SBR engagement (Zampini and D’Odorico, 2011).

SBR engagement was correlated moderately with longitudinal measures of vocabulary and was a significant predictor of scores on the EOWPVT, though this was no longer significant after controlling for age. SBR engagement was not a significant predictor of receptive vocabulary or the CDI measure of expressive vocabulary at Time 2. This pattern is consistent with previous research showing that shared reading tends to predict children’s vocabulary more strongly when measured concurrently than over longer intervals (Bus et al., 1995), largely because concurrent measures capture immediate interactional processes (Rowe, 2012). Longitudinal effects may be reduced by the high stability of vocabulary across early childhood (Bornstein et al., 2016), developmental constraints characteristic of children with DS (Fidler, 2005), and decreasing variability in shared reading practices as children become older (Deckner et al., 2006). Furthermore, long-term vocabulary growth is increasingly shaped by broader environmental inputs, such as preschool experiences and peer interaction (Hoff, 2013), which may attenuate predictive associations with early SBR engagement.

In contrast, HLE richness was not a significant predictor of vocabulary at either time point. It is worth noting however, that the correlations between HLE richness and vocabulary were moderate at Time 2 (*r’s* = 0.21–0.37) and larger than at Time 1 (*r’s* = −0.08–0.24). Environmental influences on vocabulary may become more pronounced with age, as children increasingly use and internalise language during daily interactions (Huttenlocher et al., 2010). Although rich HLEs are typically associated with stronger language development (Bus et al., 1995), the effect may not have been detected here, potentially due to the small sample size at Time 2. Prior work further suggests the possibility of lagged or cumulative effects of the HLE on vocabulary (Niklas and Schneider, 2013). Such longitudinal effects may be supported by transactional bidirectionality (Pungello et al., 2009), whereby children with stronger emerging language skills elicit and participate in more conversations, explanations, and discourse. These interactions provide additional opportunities for parental scaffolding (Lusby and Heinz, 2020), with parent behaviours adapting to children’s abilities and predicting language growth more strongly over time than concurrently (Rowe, 2012). Future studies should therefore incorporate additional follow-up assessments to evaluate whether HLE richness contributes to longer-term vocabulary development. It is also possible that the relationship between HLE and vocabulary is mediated by other factors (Muscat and Grech, 2025). For instance, while HLE richness may not directly drive vocabulary growth, it may enhance caregiver linguistic input, supporting higher-quality shared interactions that facilitate vocabulary learning (Lingwood et al., 2023).

Our study extends previous literature on toddlers (Dulin et al., 2023) by examining the relationships between aspects of the HLE and language development in young (3–6 year old) children with DS. Since vocabulary acquisition is age-related (Roberts et al., 2007), studying older children with DS may increase the likelihood of detecting associations between the HLE and vocabulary growth. Our study incorporates measures of both receptive and expressive vocabulary to account for the variable language profiles in children with DS (Roberts et al., 2007), for whom expressive vocabulary is typically relatively weaker than receptive (Seager et al., 2022). The use of distinct expressive and receptive vocabulary measures contributes to a more nuanced understanding of the variable language profiles of children with DS, and how they may be differentially impacted by HLE components. Unlike prior studies relying solely on parent-reported vocabulary assessments (Dulin et al., 2023), our study includes researcher-administered language measures to enhance reliability and validity while reducing potential biases or recall errors (Feldman et al., 2000). The longitudinal design further strengthens the study by enabling an examination of language development trajectories in relation to HLE richness and child engagement (Dulin et al., 2023).

### 4.3 Limitations and future directions

There are a few limitations to note in the current study. First, the HLE is traditionally measured in research via parent report (Dulin et al., 2023) to characterise the HLE and child engagement in SBR. Although these reports provide valuable insights, they may be less accurate than direct observational assessment data due to potential parental bias and social desirability effects. Parents may have unintentionally overestimated or incorrectly recalled (Feldman et al., 2000), the richness of the HLE or their child’s engagement. However, caregiver reports offer a broader perspective on children’s engagement, which is often variable in children with DS (Shakespeare, 2006), and thus difficult to holistically capture in a single observation. Nonetheless, future research should incorporate objective measures of the HLE, such as corroborating parent-reported data with home-based SBR observations. For example, Barton-Hulsey et al. (2020) recorded 1 to 2 h mother–child home SBR interactions, to explore maternal input and child-receptive vocabulary. Even though parents of TD children have been found to accurately report their children’s HLEs (Boudreau, 2005), refining questionnaire items could enhance measurement accuracy. Specifically, HLE richness measures should capture the *quality* of caregiver-child interactions as opposed to the mere *availability* of literacy-related activities, as interaction quality is more strongly linked to vocabulary growth (Hamilton et al., 2016). For instance, the item regarding the *quantity of books (children’s vs. adult),* and the *time spent reading* may reflect the material availability rather than the conversational or cognitive quality of the reading interactions. Although the HLE richness measure used in the current study included some items tapping interactive reading behaviours, it primarily emphasised book availability and reading frequency. This means that the “richness” score may not have fully captured the qualitative aspects of caregiver-child interaction that are most strongly related to vocabulary development (Jeremic et al., 2023). This limitation may at least partly explain why HLE richness was not a significant predictor of vocabulary in the present study, despite moderate correlations at Time 2 and despite strong theoretical support for the role of high-quality shared reading. If the measure underrepresents interaction quality, the study may underestimate the true impact of the HLE on children’s vocabulary. Future research should incorporate interaction-focussed HLE measures beyond the questionnaires, such as observing SBR interactions (Barton-Hulsey et al., 2020). Future research could also investigate whether greater engagement in structured, formal reading activities (as described in Sénéchal and LeFevre’s, 2014), combined with the maturation of children with Down syndrome, contributes to measurable gains in language proficiency. In particular, longitudinal studies could explore whether increased exposure to explicit literacy instruction, shared-book reading, and print-focused interactions supports not only vocabulary growth, but also broader aspects of language development such as morphosyntax, narrative skills, and reading comprehension as children with DS grow older.

While the sample size at Time 1 was reasonable for this population, the smaller size at Time 2 (*n* = 12) may at least partly explain the lack of significant effects (Button et al., 2013) of the HLE and child engagement on vocabulary. This may have also contributed to the larger correlations between HLE measures and vocabulary measures at Time 2 relative to Time 1. However, given the issues in recruiting large cohorts of individuals with DS, this study advances previous research based on smaller samples (e.g., Dulin et al., 2023; *n* = 13), and preliminary analyses confirmed no extreme outliers which could confound results.

An additional limitation of this study is the age range of participants. Language development varies substantially across early childhood, and the needs and abilities of younger children (e.g., around 2 years of age) may differ significantly from those of older children (e.g., up to 6 years) (van Bysterveldt et al., 2010; Ricci, 2011). This heterogeneity in developmental stages may limit the specificity and generalisability of the findings. Future research should consider examining narrower age bands or conducting age-stratified analyses to better account for developmental differences and provide more targeted insights.

Additionally, the sample predominantly contained moderate to high SES families. As SES may partially influence vocabulary development (Muscat and Grech, 2025), the results may not be generalisable to children with DS from lower SES families. Since much of the existing research on the HLEs of children with DS contains moderate to high SES families (e.g., Burgoyne and Cain, 2022), future studies should recruit more diverse samples to assess the impact of SES. Including families from lower SES backgrounds may reveal a stronger relationship between child engagement and vocabulary (e.g., Rosslund et al., 2025), as SBR could serve as a compensatory mechanism for impoverished HLEs. Given the variability in HLEs across SES groups, further research must examine how these factors shape literacy experiences with children with DS.

### 4.4 Implications and applications

Childhood educators and practitioners play a critical role in supporting caregivers of children with DS by providing strategies to promote language development (Fidler, 2005). This study highlights that child engagement during SBR is positively associated with vocabulary (Dulin et al., 2023). These findings, taken together with a large body of previous work demonstrates value in engaging children in SBR for promoting word learning (Flack et al., 2018). These results suggest, as has been noted elsewhere, that SBR is a potentially promising tool to support language development in children with DS (Jeremic et al., 2023).

Practitioners should encourage physical engagement (e.g., turning pages) and phonetic mimicry (e.g., requesting imitation) in SBR (Abbeduto et al., 2007). Supporting families to implement interactive reading techniques may foster a literacy-rich HLE and scaffold language (Barton-Hulsey et al., 2020).

Strengthening SBR in the home environment (Næss et al., 2021) aligns with family-centered intervention (Alsem et al., 2017), recognising that children’s language development is shaped by their immediate social environment (Coleman, 1988). Encouraging caregiver involvement in SBR is positively associated with increased caregiver SBR competence and child vocabulary (Dowdall et al., 2020) and should be implemented in future SBR interventions. Additionally, measuring engagement alongside vocabulary scores can refine individualised intervention targets (Bishop et al., 2016).

Taken together, these implications point to the potential value of targeting child engagement during SBR as a mechanism for supporting expressive vocabulary development in children with DS. However, to establish whether enhancing engagement *causes* improvements in language outcomes, this association needs to be tested rigorously through controlled intervention studies (e.g., Burgoyne et al., 2023). Future research should therefore examine whether programmes that explicitly teach caregivers to use engagement-focused strategies, such as interactive prompts, gesture-supported reading, or materials designed to elicit participation, lead to greater vocabulary gains compared with standard SBR practices. Randomised or quasi-experimental designs comparing “engaging” reading interventions with typical reading experiences would allow researchers to determine whether increased engagement drives measurable improvements in expressive vocabulary over time. Such studies are essential for clarifying the causal role of engagement, refining evidence-based recommendations for practitioners, and developing targeted reading interventions tailored to the learning profiles of children with DS.

These findings are increasingly relevant as the lifetime prevalence of DS is rising due to improved life expectancy (Wu and Morris, 2013). Scalable, low-cost interventions aimed at increasing engagement in SBR could potentially help address language delays seen in DS, increasing access to inclusive education for individuals with DS (Hughes, 2006). Enhancing early language development may promote academic success, social participation and quality of life in these individuals (Stojanovik et al., 2024) and potentially decrease the reliance on specialised educational resources.

## Data Availability

The raw data supporting the conclusions of this article will be made available by the authors, without undue reservation.
