# Supplementary material for: Concurrent and longitudinal associations between the Home Literacy Environment and the language skills of children with Down syndrome
Source: Front Psychol. 2026 Apr 28;17:1795715. doi: 10.3389/fpsyg.2026.1795715 (PMC13160870; doi:10.3389/fpsyg.2026.1795715)
Supplement: Supplementary file 1 [file Supplementary_file_1.docx]

**Appendix A. Scoring for HLE items**

**HLE Richness Composite**

| **HLE Questionnaire Item** | **Code and Points** |
| --- | --- |
| How often parent reads for enjoyment (Q3a) | Never: 0; 1-2 times: 1; 3-4 times: 2; 5-6 times: 3; 7-8 times: 4; 9-10 times: 5; 11+ times: 6 |
| How often parent reads informative books (Q3b) | Never: 0; 1-2 times: 1; 3-4 times: 2; 5-6 times: 3; 7-8 times: 4; 9-10 times: 5; 11+ times: 6 |
| Y/N Designated reading time (Q6) | Yes: 1; No: 0 |
| Times during week read to child (Q7) | Never: 0; 1-2 times: 1; 3-4 times: 2; 5-6 times: 3; 7-8 times: 4; 9-10 times: 5; 11+ times: 6 |
| Number of books read to child during week (Q8) | N/A: 0; 1-2 books: 1; 3-4 books: 2; 5-6 books: 3; 7-8 books: 4; 9-10 books: 5; 11+ books: 6 |
| Number of books read to child in one sitting (Q9) | N/A: 0 ; 1-2 books: 1; 3-4 books: 2; 5-6 books: 3; 7-8; books: 4; 9-10 books: 5; 11+ books: 6 |
| Time reading to child in one sitting (Q10) | < 10 mins: 1; 10- 20 mins: 2; 21- 30 mins: 3; 31- 40 mins: 4; 41- 50 mins: 5; 51- 60 mins: 6; < 1 hr: 7 |
| Time per week reading to child (Q11) | < 15 mins: 1; 15- 30 mins: 2; 30- 45 mins: 3; 1- 2 hrs: 4; 3- 4 hrs: 5; 5- 6 hrs: 6; 7+ hrs: 7 |
| Agree/Disagree: Point out details while reading (Q12a) | Strongly Disagree: 1; Disagree: 2; Agree: 3; Strongly Agree: 4 |
| Agree/Disagree: Relate story events to child’s interactions (Q12b) | Strongly Disagree: 1; Disagree: 2; Agree: 3; Strongly Agree: 4 |
| Agree/Disagree: Ask questions about story and follow-up with answers (Q12c) | Strongly Disagree: 1; Disagree: 2; Agree: 3; Strongly Agree: 4 |
| Agree/Disagree: Teach letters/sounds while reading (Q12d) | Strongly Disagree: 1; Disagree: 2; Agree: 3; Strongly Agree: 4 |
| Times visited library (Q14) | Never: 0; Once: 1; Every 4-6 months: 2; Every 2-3 months: 3; Monthly: 4; Every 2-3 weeks: 5; Weekly: 6 |

A composite for parent-reported richness of the HLE incorporated thirteen questions regarding the quality and quantity of literacy-related activities which the child was exposed to within the family home. The sum of these thirteen items indicated the overall quality of the HLE, with higher scores indicating richer HLEs (maximum score: 67).

**Child Engagement Composite**

| **HLE Questionnaire Item** | **Code and Points** |
| --- | --- |
| **Times your child asks to read (Q16a)** | Never: 0; 1-2 times: 1; 3-4 times: 2; 5-6 times: 3; 7-8 times: 4; 9-10 times: 5; 11+ times: 6 |
| **Times your child pretends to read (Q16b)** | Never: 0; 1-2 times: 1; 3-4 times: 2; 5-6 times: 3; 7-8 times: 4; 9-10 times: 5; 11+ times: 6 |
| **Grabs book during reading (Q17a)** | Never: 0; Has but rarely: 1; Occasionally: 2; A few times per story: 3; Very frequently during story: 4 |
| **Turns pages during reading (Q17b)** | Never: 0; Has but rarely: 1; Occasionally: 2; A few times per story: 3; Very frequently during story: 4 |
| **Points to pictures or words during reading (Q17c)** | Never: 0; Has but rarely: 1; Occasionally: 2; A few times per story: 3; Very frequently during story: 4 |
| **Names pictures during reading (Q17d)** | Never: 0; Has but rarely: 1; Occasionally: 2; A few times per story: 3; Very frequently during story: 4 |
| **Asks questions about characters/events during reading (Q17e)** | Never: 0; Has but rarely: 1; Occasionally: 2; A few times per story: 3; Very frequently during story: 4 |
| **Fills in words/lines during reading (Q17f)** | Never: 0; Has but rarely: 1; Occasionally: 2; A few times per story: 3; Very frequently during story: 4 |

A composite for child engagement during shared storybook reading consisted of eight questions related to what the child does during a typical, shared book reading interaction. The sum of these eight items indicated children’s engagement in reading, with higher scores indicating greater engagement in shared storybook reading (maximum score: 36).
